# Improvement of irritable bowel syndrome with glucagon like peptide-1 receptor agonists: a systematic review and meta-analysis

**DOI:** 10.3389/fendo.2025.1548346

**Published:** 2025-03-11

**Authors:** Mohamed E. A. Mostafa, Tariq Alrasheed

**Affiliations:** ^1^ Department of Anatomy, Faculty of Medicine, University of Tabuk, Tabuk, Saudi Arabia; ^2^ Department of Internal Medicine, Faculty of Medicine, University of Tabuk, Tabuk, Saudi Arabia

**Keywords:** glucagon like peptide-1, irritable bowel syndrome, pain, IBS, agonist

## Abstract

**Introduction:**

Irritable bowel syndrome (IBS) is a severe gastrointestinal condition with symptoms like pain, bloating, diarrhea, and constipation. Glucagon-like peptide-1 (GLP-1) receptors, expressed in the central nervous system and peripheral tissues, have been found to affect gut motility. GLP-1 and its analog ROSE-010 have been shown to inhibit the migrating motor complex and decrease gastrointestinal motility in IBS patients.

**Aim:**

This systematic review and meta-analysis aim to assess the efficacy and safety of GLP-1 receptor agonists in providing pain and symptom relief for individuals with IBS.

**Methods:**

The study conducted extensive searches across various databases, including Cochrane Library, Web of Science, PubMed, Google Scholar, and Science Direct, to identify studies on IBS and related drugs. A search strategy using keywords and medical subject heading terms (MeSH) was developed to ensure inclusivity. Exclusion criteria included non-English language studies, books, conference papers, case reports, *in vitro* studies, animal studies, and non-original articles.

**Results:**

The study found that ROSE-010 (100 µg) significantly lowered pain intensity in IBS patients compared to a placebo, with an overall odds ratio of 2.30, 95% CI: 1.53-3.46. ROSE-010 (300 µg) is more effective than a placebo for all irritable bowel syndrome subtypes, with consistent effects across trials. ROSE-010 is linked to a greater incidence of nausea, vomiting, and headache than placebo.

**Conclusion:**

ROSE-010, a glucagon-like peptide-1 receptor agonist, has been shown to reduce pain in individuals with IBS. However, its higher frequency of nausea, vomiting, and headache suggests the need for close monitoring and individualized treatment plans. Further investigation is needed to understand its impact on different IBS subtypes and long-term effects.

**Systematic review registration:**

https://www.crd.york.ac.uk/PROSPERO/, identifier CRD42024613545.

## Introduction

Irritable bowel syndrome (IBS) is a prevalent and debilitating gastrointestinal condition characterized by recurrent flare-ups of symptoms such as increased central pain, bloating, diarrhea, and constipation (1, 2). The condition can significantly lower the quality of life for those affected, and diagnosis is based on symptoms, making it challenging to diagnose (3). Risk factors for IBS include childhood trauma, family history of IBS, being female, and having a past gastrointestinal (GI) infection (4). IBS patients have increased levels of mast cells, lymphocytes, and mucosal T cells, which suggests an immunological activation involvement (5, 6).

Post-viral gastroenteritis, pre-morbid psychological disorders, and the hypothalamic-pituitary-adrenal axis mediate a maladaptive stress response, which is essential for the onset, intensity, and persistence of IBS-associated symptoms (7). IBS patients are also more likely to co-morbid with mood disorders like anxiety and depression (8, 9).

The Rome IV diagnostic criteria are currently used to diagnose IBS, which is divided into four subgroups based on bowel habits, bowel function, and stool consistency: constipation dominant (IBS-C), diarrhea dominant (IBS-D), mixed (IBS-M), and unclassified/unspecified IBS (IBS-U) (10). IBS-related pain is thought to be caused by lowered sensory threshold and disturbed smooth muscle activation, leading to visceral hypersensitivity. IBS is difficult to treat, with few practical therapy approaches available despite its high incidence, financial and health costs, and severity (3). Relieving symptoms and enhancing quality of life are the primary therapy goals (11).

Following food consumption, the metabolic endocrine cascade begins with gastric emptying. Insulin secretion is stimulated, glucagon and gastric acid secretion are inhibited, and GI transit and motility are decreased by incretin hormones, especially glucagon-like peptide-1 (GLP-1), which is released postprandially from L-cells lining the gut in response to food ingestion (12). Furthermore, both *in vitro* and *in vivo*, GLP-1 has been frequently demonstrated to reduce GI muscle activity via nerve-mediated mechanisms reliant on nitric oxide (13, 14).

GLP-1 receptors, which are expressed in the central nervous system (CNS) in addition to peripheral tissues, are limited to neurons in the caudal nucleus of the solitary tract (NTS) and the ventrolateral medulla in the brainstem and hypothalamus (15–17). Biologically active GLP-1 has a high affinity for this receptor. Additionally, the GLP-1receptors have been identified in the GI tract’s myenteric and submucosal neural plexuses (18, 19). Several investigations have demonstrated that GLP-1 and its particular analog ROSE-010 have a significant effect on the gut’s motility pattern (13, 20, 21). In both healthy individuals and IBS patients, GLP-1 inhibited the migrating motor complex (MMC) and decreased motility in the antro-duodeno-jejunal area (22, 23). Giving the GLP-1 analogue ROSE-010 to a mixed group of IBS patients decreased GI motility and relieved acute discomfort, according to a placebo-controlled double-blind crossover clinical experiment (24).

Constipation-predominant IBS was linked to lower mucosal expression of GLP-1 receptors and serum GLP-1 concentrations (25, 26). The notion that reduced GLP-1 concentrations would result in a reduction of the prokinetic actions of GLP-1 in the colon (27), causing constipation and abdominal pain, was further supported by the correlation between this and the intensity of abdominal discomfort (28). A rat model of visceral pain sensitivity also showed a decrease in circulating amounts of bioactive GLP-1 (23). A previous study revealed that GLP-1 and ROSE-010 suppress postprandial GI motility, most likely via GLP-1 receptors at myenteric neurons, necessitating cyclic adenosine monophosphate (cAMP) and functional nitrergic signaling (13). Although ROSE-010 administration is usually well tolerated, adverse events (AEs) exist (29). Rarely are the drug’s anticipated side effects which include headache, nausea, vomiting, and decreased blood glucose (30–32).

This is the first systematic review and meta-analysis investigating GLP-1 agonists’ efficacy and safety in IBS patients. This review synthesizes existing evidence, assesses efficacy and safety, and strengthens the evidence base, ultimately improving patient care and optimizing IBS treatment techniques. The systematic review and meta-analysis aim to assess the efficacy of GLP-1 R agonist medicines in providing pain and symptom relief for individuals with IBS. Furthermore, the review aims to determine the incidence of adverse effects associated with GLP-1 agonist medications against placebo or standard of therapy.

## Methods

Under registration number CRD42024613545, the research protocol for this study was submitted to the International Prospective Register of Systematic Reviews (PROSPERO). To guarantee a methodical approach to the search process and reporting of the results displayed in Supplementary Appendix **S1**, the Preferred Reporting Items for Systematic Reviews and Meta-Analyses (*PRISMA*) checklist criteria were adhered to (33).

First, extensive searches were carried out across a number of databases in the Cochrane Library, Web of Science, PubMed, Google Scholar, and Science Direct. After that, a screening of the identified studies’ titles and abstracts was conducted. To find any pertinent papers, a manual search was also conducted through the reference lists of the included research. After that, the full texts were evaluated considering the preset inclusion and exclusion standards.

Discussions were performed to settle any disputes or inconsistencies, and the original author made the ultimate decision. The included studies were then subjected to a quality assessment. The findings were then combined, and meta-analyses were performed to examine and interpret the results.

### Search strategy

We used the following search strategy: (“irritable bowel syndrome” OR “IBS” OR “spastic colon” OR “irritable colon”) AND (“Glucagon like peptide 1 receptor agonist” OR “GLP-1 agonist” OR “Semaglutide” OR “Dulaglutide” OR “Exenatide” OR “Liraglutide” OR “Lixisenatide” OR “Tirzepatide”). Electronically, a thorough investigation was carried out on 5 distinct databases—PubMed, Google Scholar, Science Direct, Web of Science, and Cochrane Library—during the period from 20 October to 15 November 2024. A search strategy using a combination of keywords and medical subject heading terms (MeSH) was developed to guarantee inclusiveness.

### Participants and requirements for inclusion

To determine whether papers were appropriate for inclusion in our analysis, a screening process based on the PICOS (population, interventions, comparators, outcomes, and study designs) formatting style was used as shown in **Table 1**.

**Table 1:** PICOS criteria of the included studies.

Criteria	Inclusion
Population	Patients of any age diagnosed with irritable bowel syndrome (IBS)
Intervention	Glucagon like peptide 1 receptor agonist drugs
Comparator	Placebo or standard of care
Outcome	• Primary outcomes: Pain and symptoms of IBS relief • Secondary outcomes: Frequency of adverse effects related to GLP-1 agonists
Study design	Randomized and non-randomized controlled trials.

The study aimed to include patients diagnosed with irritable bowel syndrome, original articles, and no restrictions on study design, country, socioeconomic status, or publication year. Exclusion criteria included unreliably extracted data, non-English language studies, books, conference papers, case reports, and articles without full text, and non-original articles like reviews and meta-analyses.

### Extraction of data

Data from the trials, including the name of the principal investigator, the year of publication, the sample size, and the research design, were extracted using a pre-made template. Furthermore, we considered the patients’ baseline data, which included age, sex, and inclusion and exclusion criteria. In addition, we extracted the type, dose, route, duration, and outcomes of GLP-1 administration. Data were independently extracted in duplicate by two investigators, and disagreements were settled by consensus.

### Evaluation of bias risk

The Cochrane revised tool for assessing risk of bias in randomized trials (RoB 2) was utilized to critically assess the papers that were part of our investigation (34). Each study was assessed by two independent reviewers in five areas: (1) bias resulting from the randomization procedure; (2) bias resulting from deviations from intended interventions; (3) bias resulting from missing outcome data; (4) bias in outcome assessment; and (5) bias in the selection of the reported result. A third reviewer was consulted or discussed in order to address any discrepancies. Each study’s overall risk of bias was classified as low, some concerns, or high. A risk of bias summary and graph were used to display the findings. The case-control study was evaluated using the Critical Appraisal Skills Program (CASP) version for case-control studies (35). The assessment results are available in the **Supplementary Appendix S2, S3**.

### Data analysis and heterogeneity assessment

R version 4.2.1 was used to conduct the meta-analysis. Package “meta” was used for conducting the analysis which is a very useful tool for performing meta-analyses. It provides a number of functions for running several sorts of meta-analyses, including fixed-effect and random-effects models, as well as forest plots and funnel plots. The package supports a variety of effect measures, including the odds ratio, risk ratio, risk difference, mean difference, and standardized mean difference. It also offers statistics to measure heterogeneity.

Study-specific OR estimates were pooled using either a fixed-effects model or a random-effects model in case a significant heterogeneity exists. Heterogeneity between studies was evaluated using the Cochran’s Q and I^2^ tests. A fixed-effect model was used for meta-analysis where heterogeneity was less than 50%. When heterogeneity was greater than 50%, a random-effects model was employed.

## Results

### Studies selection

**Figure 1:**
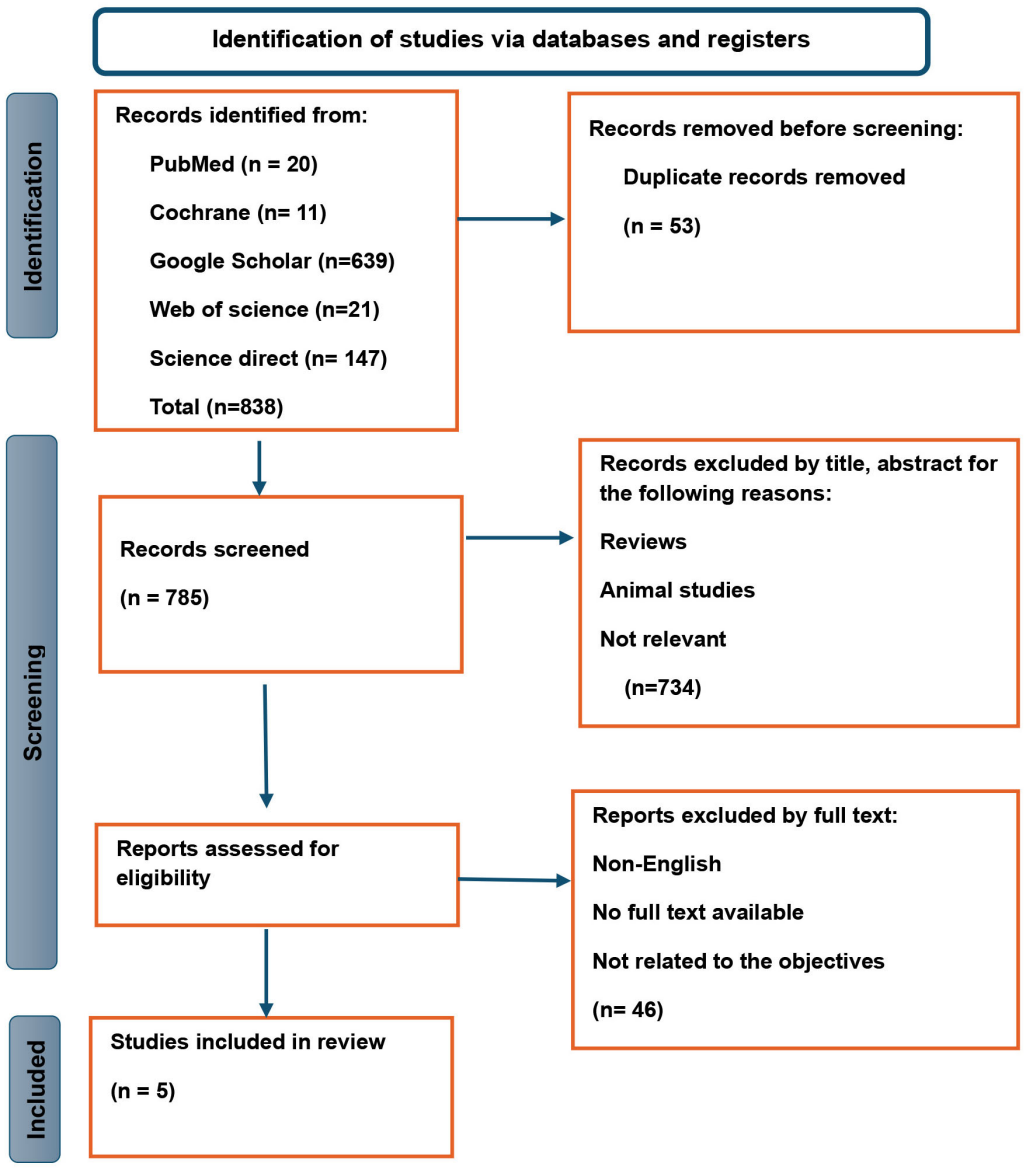
PRISMA flow diagram for systematic reviews.

Our search identified 838 records in total from different databases. Duplicates identified were 53, 734 records were excluded by screening title and abstract. The remaining 51 records were retrieved in full text for eligibility assessment. After careful review, 5 studies were found eligible for the review (**Figure 1**).

### Characteristics of the included studies

**Table 2** summarizes the characteristics of studies assessing the effects of the GLP-1 agonist ROSE-010 on IBS patients. The studies employed randomized, double-blind, placebo-controlled designs except Li (2017) conducted a case-control study comparing patients with IBS-C to a control group without IBS-C. The total sample size in all the studies was 476 and varied greatly, with smaller studies having 38 participants and larger ones having 166 each. The studies were conducted in a variety of countries (USA, Sweden, Germany, Denmark, and China), which increased the generalizability of the findings across populations. Most studies included multiple doses of ROSE-010 and a placebo group. In crossover designs, individuals received both placebo and active therapy at various times.

**Table 2:** Characteristics of the included studies.

Study ID	Country	Study design	Total sample size	Sample size in each group	Quality assessment
Camilleri, 2012 (21)	USA	A single-center, randomized, parallel-group, double-blind, placebo-controlled trial	46	30 µg: 11 100 µg: 11 300 µg: 12 placebo: 12	Some concern
Hellström, 2008 (22)	Sweden	A single-center, randomized, placebo-controlled study	38	Group I: 6 Group II: 8 Group III: 8 Group IV:8 Group V: 8	Some Concern
Hellström, 2009 (24)	Germany, Sweden, and Denmark.	A randomized, placebo-controlled, double-blind, crossover study	166	Placebo: 125 ROSE-010 at 100 µg: 134 ROSE-010 at 300 µg: 128	Some concern
Li, 2017 (28)	China	Case-control study	60	Patients: 38 Control: 22	Moderate risk
Touny, 2022 (12)	Sweden	Double-blind, randomized, cross-over design	166	Placebo: 125 ROSE-010 100 mg: 134 ROSE-010 300 mg: 128	Some concern

### Characteristics of GLP-1 agonist and control

**Table 3** highlights important details such as dosage, delivery techniques, control groups, and follow-up periods while summarizing research examining the effects of the GLP-1 agonist ROSE-010. The findings indicate the following:

**Table 3:** Characteristics of GLP-1 agonist and control.

Study ID	Name of GLP-1 agonist	Dose	Route of administration	Frequency, duration	Description of control group	Total follow-up duration
Camilleri, 2012 (21)	ROSE-010	30, 100, and 300 µg	Subcutaneous	Once daily for 3 consecutive days and on 1 day 2–10 days later	A matching placebo via abdominal subcutaneous injection once daily for 3 consecutive days.	36 days
Hellström, 2008 (22)	ROSE-010	Group II: 0.7 pmol/kg/min Group III: 1 1.2 pmol/kg/min Group IV: 1.2 pmol/kg/min Group V: 2.5 pmol/kg/min	Intravenous	Infusion for 4 hours after 4 hours of intravenous saline	Had no gastrointestinal symptoms or disease and had not had abdominal surgery	
Hellström, 2009 (24)	ROSE-010	100 µg and 300 µg	Subcutaneous	Single SC injection	placebo treatment in a crossover	For 24 hours and 4 weeks after the last treatment visit
Li, 2017 (28)					consisted of 22 healthy subjects who underwent colonoscopy for polyp or cancer surveillance, with all participants having negative results. They were matched for age and gender with the IBS-C group	
Touny, 2022 (12)	ROSE-010	100 µg and 300 µg	Subcutaneous	Single SC injection	0.3 mL isotonic saline solution via subcutaneous injection, which was visually identical to the ROSE-010 injections to maintain blinding.	Maximal period of three months, with a minimum of 24 h between each study drug administration

Doses and Administration: The studies examined a range of ROSE-010 doses (from 30 µg to 300 µg) administered by subcutaneous and intravenous methods. The therapies were administered as single injections or infusions, and the regimens varied from study to study, ranging from a single dose to daily administration for a few days.

Control Groups: To ensure blinding and reduce bias, the majority of trials compared ROSE-010 to placebos (either saline or identical injections). To improve comparability, some studies carefully matched the control groups to the treatment groups based on gender and age (Li, 2017).

Follow-Up Duration: From a few hours to several months, the follow-up duration varied greatly among the studies. While Camilleri (2012) and Touny (2022) used prolonged monitoring periods of weeks or months to evaluate long-term effects, Hellström (2008) study had no follow-up following the treatment period.

### Characteristics of the studies’ participants

**Table 4** summarizes the characteristics of the participants in the included studies. Participants ranged in age from 18 to 70 years, with females surpassing males in most trials. The inclusion criteria included being diagnosed with IBS and suffering abdominal pain or discomfort. Exclusion criteria included pregnancy, lactation, recent abdominal surgery, structural abnormalities, a history of organic diseases, severe metabolic disorders, or GI-related drugs. To reduce confounding effects, some studies excluded participants who were taking certain drugs or had specific GI conditions other than IBS.

**Table 4:** Characteristics of the studies’ participants.

Study ID	Age of participants	Sex of participants	Inclusion criteria	Exclusion criteria
Camilleri, 2012 (21)	18- 65 years	Females	The study involved female participants aged 18-65 years with a previous diagnosis of IBS, a normal rectal examination within the past two years, and no evacuation disorder, such as high sphincter tone, failure of perineal descent, or spasm.	The study excluded women who were pregnant or breastfeeding, had significant abnormal physical examinations or laboratory results, had structural or metabolic diseases affecting the GI system, or had difficulty withdrawing medications affecting GI transit 48 hours prior to the study.
Hellström, 2008 (22)	IBS 22-59 years, controls 18-55 years	Controls: all males, IBS: 3 males, 13 females	Rome II criteria for IBS of both the Diarrhea and constipation Not mentioned predominant type	
Hellström, 2009 (24)	42.4 ± 15.0 years	70% females, 30% males	The study focuses on individuals aged 18-70 with IBS who experience at least four abdominal pain attacks per month for at least two months, lasting at least two hours and with pain intensity of 40 mm or higher.	Patients with a history of disease, condition, or medication interference; recent abdominal surgery; gastrointestinal tract abnormalities; pregnant or breastfeeding women; and those consuming less than 3 hours before the visit.
Li, 2017 (28)	IBS-C (mean 48.5 years, healthy controls (mean, 48.1 years	IBS-C; 21 females and 17 males) and healthy controls (13 females and 9 males)	Patients experienced abdominal pain or discomfort for at least 3 days every month in the last 3 months, with a duration of at least 6 months. They had loose or watery stools less than 25% of the time, hard or lumpy stools 25% or more, and symptoms associated with bowel movement alterations.	Participants with organic diseases like asthma, celiac, colon, and gastrointestinal issues, as well as females with associated symptoms like irritable bladder, dysmenorrhea, chronic pelvic pain syndrome, and painful gynecologic disorders.
Touny, 2022 (12)	18-70 years	116 females, 50 males Not mentioned Not mentioned		

### Outcomes of GLP-1 agonists administration

The studies’ findings indicated that ROSE-010 and GLP-1 have prospective therapeutic effects on the management of IBS, particularly in subtypes defined by constipation (IBS-C) and mixed symptoms, as shown in **Table 5**.

**Table 5:** Outcome assessment and conclusions.

Study ID	Method of IBS diagnosis	Outcomes	Conclusion
Camilleri, 2012 (21)	Rome III criteria	The study found that ROSE-010 significantly retarded gastric emptying but did not significantly affect gastric volumes, small bowel or colonic transit, or bowel functions. The 30- and 100-g doses accelerated colonic transit at 48 hours, with no clinically significant safety results.	ROSE-010 delayed gastric emptying in IBS-C but did not alter colonic transit or accommodation. It accelerated colonic transit at 48 hours with 30 and 100 g, suggesting potential constipation relief.
Hellström, 2008 (22)	Rome II criteria	GLP-1, a hormone, can reduce migrating motor complexes (MMCs) in healthy subjects and IBS patients. In healthy subjects, it reduced MMCs from 2 to 0.5 and motility index from 4.9 to 4.3 ln P (mmHg*s min)1). In IBS patients, it reduced MMCs from 2.5 to 1 without affecting the motility index. At 2.5 pmol kg)1 min)^-1^, it decreased MMCs from 2 to 1 and the motility index. Motility responses were similar in both the antrum and duodenum.	The gut peptide GLP-1 decreases motility in the antro-duodeno-jejunal region and inhibits the MMC in healthy subjects and IBS patients, as confirmed by reverse transcriptase PCR.
Hellström, 2009 (24)	The Rome criteria for IBS diagnosis include abdominal pain or discomfort for at least 3 days per month in the last 3 months, improvement with defecation, onset associated with stool frequency and form, and no demonstrable mechanical, inflammatory, or biochemical causes.	The study found that ROSE-010 injections resulted in twice as many patients responding to the primary efficacy endpoint compared to placebo (24%, 23%, and 12% after 300 µg, 100 µg, and placebo injections, respectively). The times to meaningful and total pain relief were shorter for both doses. More patients were satisfied with ROSE-010 than previous IBS medications.	ROSE-010 was well tolerated and provided fast and effective relief of acute pain attacks on demand in IBS patients
Li, 2017 (28)	IBS-C diagnosis is based on ROME III criteria, which include specific symptom patterns. Patients must experience abdominal pain or discomfort for at least 3 days per month in the last 3 months, with hard or lumpy stools occurring 25% or more.	The study found that patients with IBS-C had significantly decreased serum GLP-1, which negatively correlated with abdominal pain scores. Biopsies revealed a significant down-regulation of the GLP-1 receptor in colonic mucosa. The study evaluated the efficacy and safety of ROSE-010 in alleviating IBS symptoms.	The study suggests that decreased serum GLP-1 levels may be linked to abdominal pain in patients with IBS-C, potentially alleviated by ROSE-010.
Touny, 2022 (12)	Rome criteria	The study found that ROSE-010 provided significant pain relief at a dose of 300 mg compared to 100 mg and a placebo at 120 minutes’ post-injection. Females experienced greater pain relief than males, and age and BMI did not affect treatment response. The pain relief was most effective in patients with constipation-dominant and mixed IBS. Responders were those who achieved at least a 50% reduction in pain intensity within 1-hour post-treatment. Common adverse events included nausea, hypoglycemia, and dyspepsia, with higher incidence at the 300 mg dose.	Clinical trial data shows female participants respond more to ROSE-010 for IBS pain relief, with maximum relief achieved at 120 minutes with higher doses but higher nausea rates. IBS-C and IBS-M showed the most significant improvement in pain attacks.

Pain relief: ROSE-010 consistently offered significant, quick pain alleviation in IBS patients, with increased efficacy at higher doses (300 mg), and was more effective in females and IBS-C/IBS-M subtypes. ROSE-010 delayed gastric emptying in some patients but also increased colonic transit at larger doses, perhaps improving constipation resolution in IBS-C.

GLP-1 Impact: GLP-1 has been proven to lower motility in certain parts of the digestive system (such as the antrum and duodenum), which may help relieve IBS symptoms. IBS-C patients had lower serum GLP-1 levels, which was connected with abdominal pain, indicating GLP-1’s potential for alleviating pain in these patients. Safety and Side Effects: ROSE-010 was generally well tolerated; however, larger doses were associated with greater side effects, primarily nausea.

### Quality assessment of the included studies

**Figure 2:**
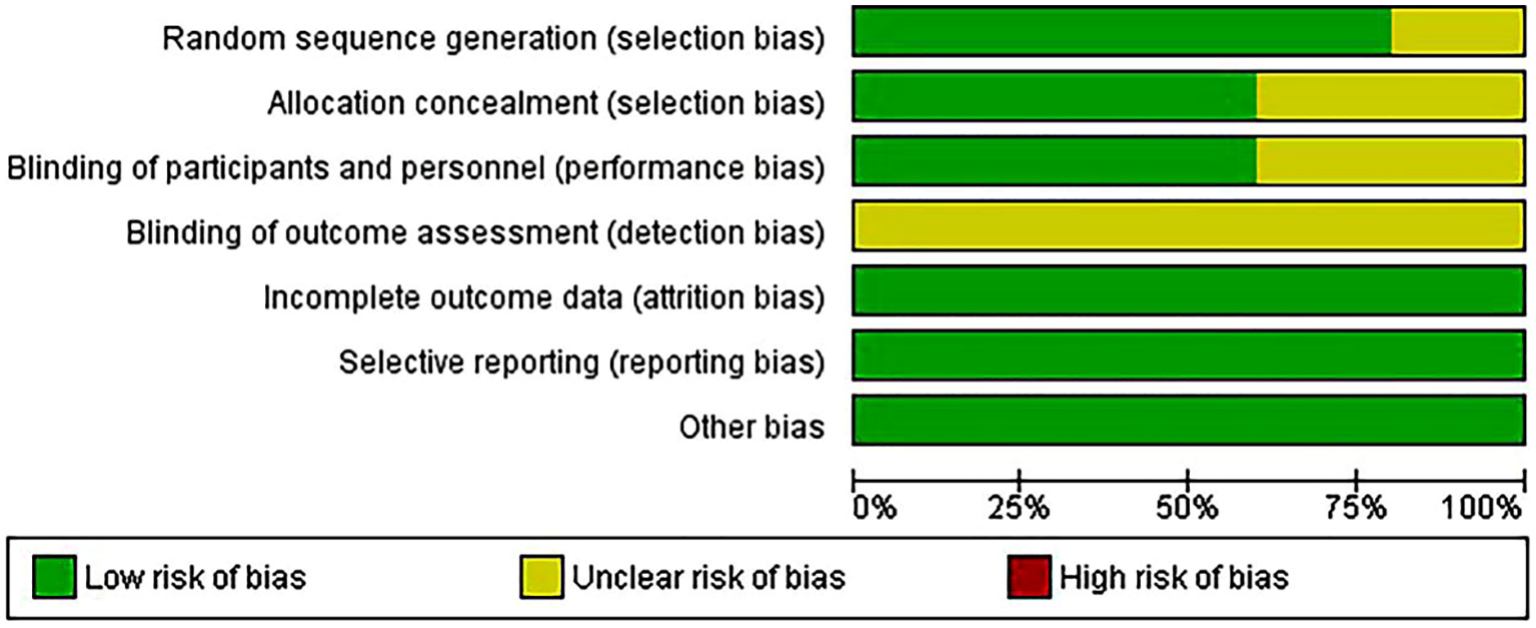
Risk of bias graph.

**Figure 3:**
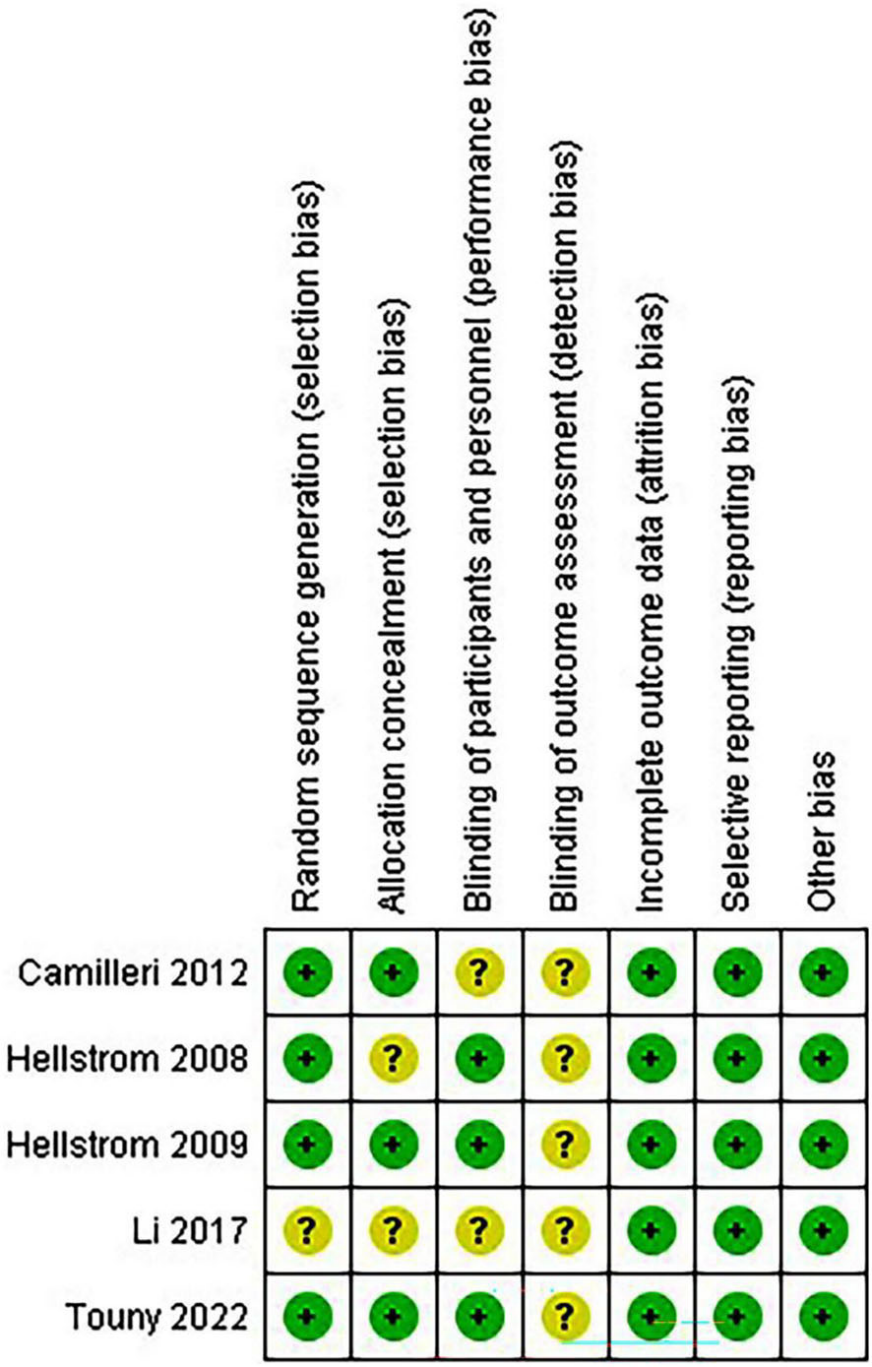
Risk of bias summary.

The risk of bias in random sequence generation and allocation is low in most trials, while the risk of participant and staff blinding varies. Additionally, blinding for outcome evaluation entails a variety of bias risks. Most studies have a low risk of bias in selective outcome data (**Figures 2**, **3**).

### Meta-analysis of the outcome

#### Pain relief with GLP-1 agonists versus placebo

**Figure 4:**
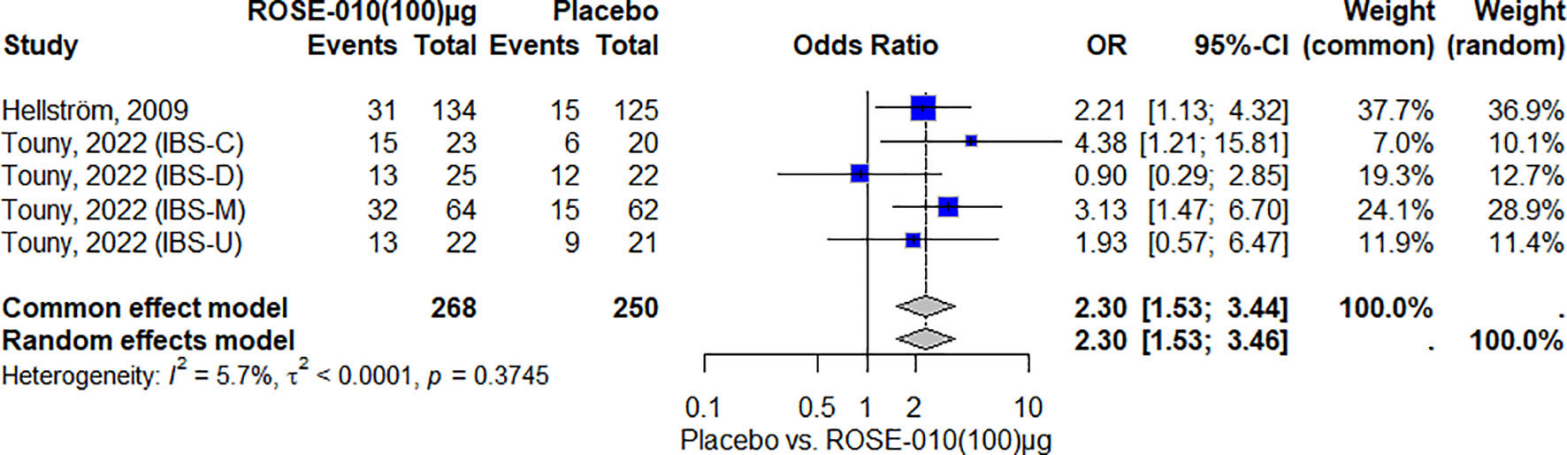
Effect of ROSE-010 (100 µg) versus placebo on pain relief.

According to the results, ROSE-010 (100 µg) considerably lowers the pain intensity when compared to a placebo. The effect is largely consistent throughout the included trials, as indicated by the low heterogeneity. With an overall odds ratio of 2.30, ROSE-010 appears to have significant benefits over a placebo (**Figure 4**).

**Figure 5:**
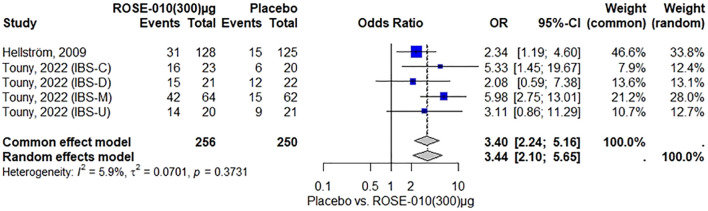
Effect of ROSE-010 (300 µg) versus placebo on pain relief.

According to the findings, ROSE-010 (300 µg) is considerably more effective than a placebo for all irritable bowel syndrome subtypes in relieving the pain. The effect is largely consistent throughout the included trials, as indicated by the low heterogeneity. Overall odds ratios from the random effects model (3.44) and common effect model (3.40) indicate that ROSE-010 is significantly better than a placebo (**Figure 5**).

#### Adverse effects associated with GLP-1 agonist and control

##### Nausea

**Figure 6:**
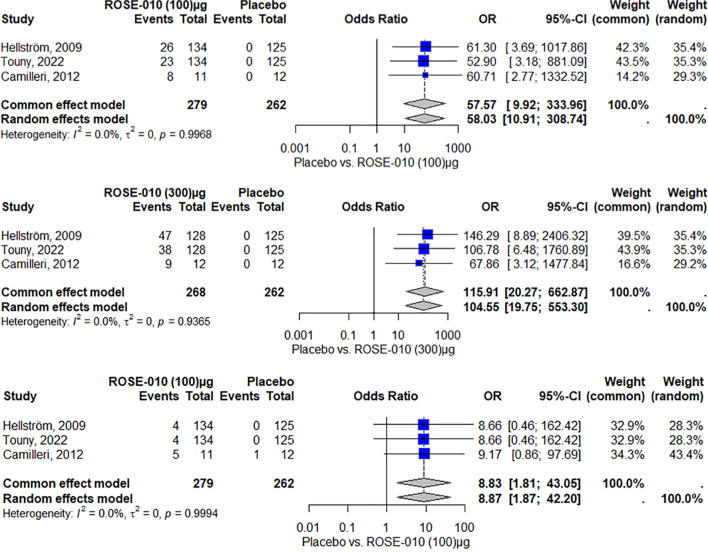
Nausea occurrence with ROSE-010 (100 µg) versus placebo. Nausea occurrence with ROSE-010 (300 µg) versus placebo. Vomiting occurrence with ROSE-010 (100 µg) versus placebo.

The findings indicate that, among the included studies, ROSE-010 (100 µg) is substantially linked to an increased risk of nausea. The effect is consistent throughout the investigations, as evidenced by no heterogeneity (I^2^ = 0.0%). With high odds ratios and large confidence intervals that suggest reduced precision because of the small sample size, the overall odds ratios from the random effects model (58.03) and the common effect model (57.57) both indicate a higher incidence of nausea over placebo (**Figure 6**).

**Figure 7:**
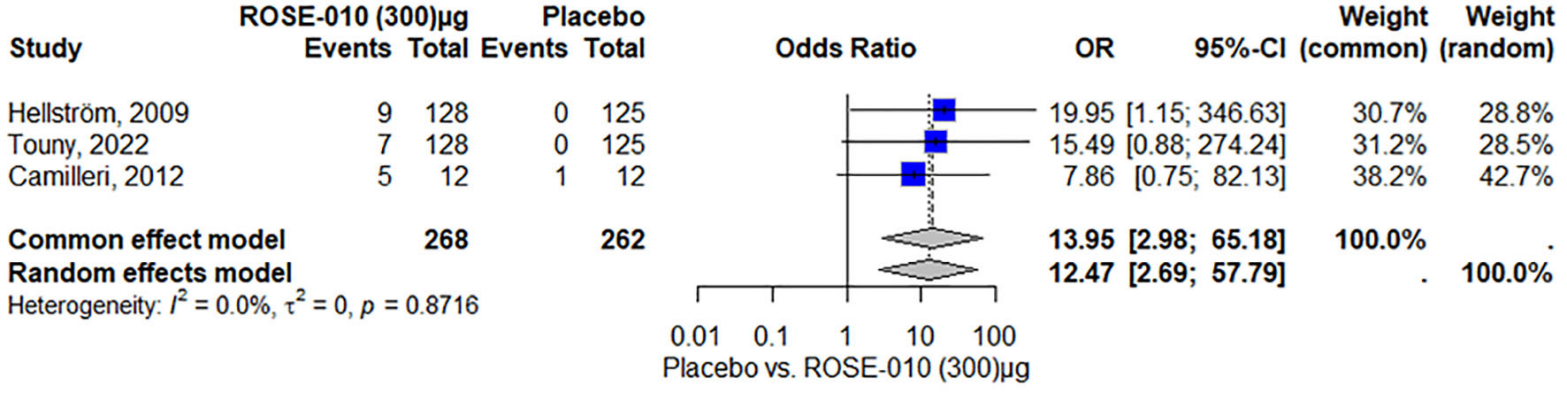
Vomiting occurrence with ROSE-010 (300 µg) versus placebo.

ROSE-010 (300 µg) is substantially linked to a higher incidence of nausea when compared to a placebo. The effect is consistent throughout the investigations, as evidenced by no heterogeneity (I^2^ = 0.0%). With high odds ratios and wide confidence intervals, the overall odds ratios from the random effects model (104.55) and the common effect model (115.91) demonstrate a significant increase in the incidence of nausea with ROSE-010 administration (**Figure 7**).

##### Vomiting

According to the results, vomiting is more common in the treatment group (ROSE-010 at 100 µg) than in the placebo group in all of the included investigations. Both the common effect model (8.83) and the random effects model (8.87) have odds ratios (OR) that show a statistically significant increase in vomiting in the treatment group. The results appear to be consistent throughout the investigations, as indicated by the minimal heterogeneity (I^2^ = 0.0%) as shown in **Figure 7**.

**Figure 8:**
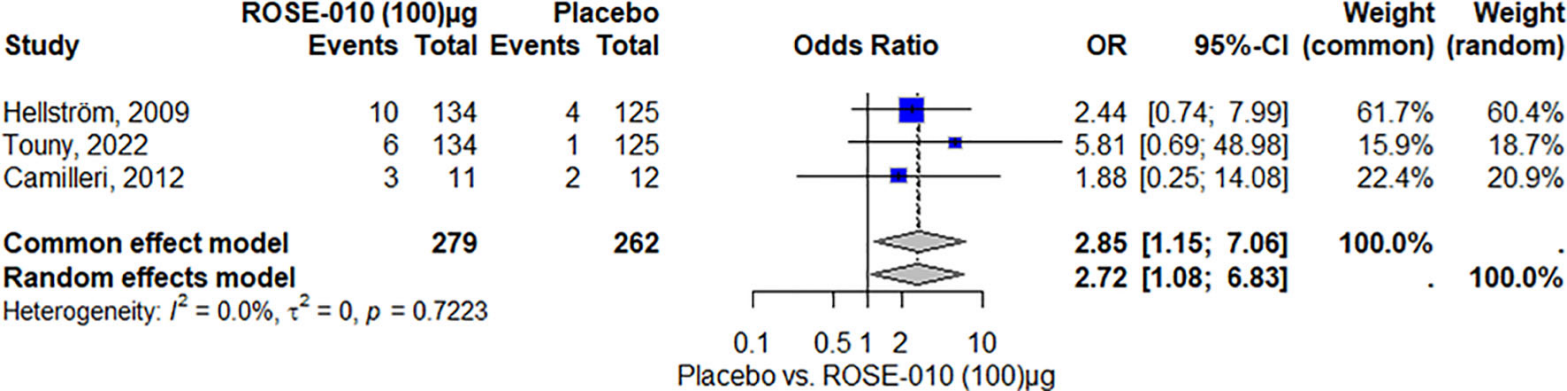
Headache occurrence with ROSE-010 (100 µg) versus placebo.

Vomiting is more common in the treatment group (ROSE-010 at 300 µg) than in the placebo group in all of the included investigations. Both the random effects model (12.47) and the common effect model (13.95) have odds ratios (OR) that show a statistically significant increase in vomiting in the treatment group. The results appear to be consistent throughout the investigations, as indicated by the minimal heterogeneity (I^2^ = 0.0%) as shown in **Figure 8**.

##### Headache

**Figure 9:**
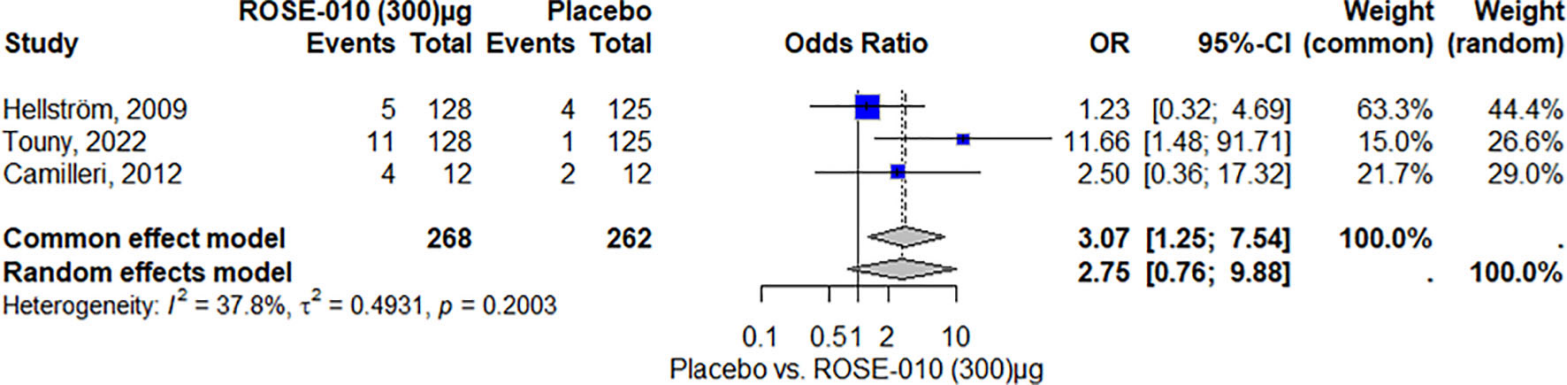
Headache occurrence with ROSE-010 (300 µg) versus placebo.

According to the study, headaches were more common in the ROSE-010 treatment group (100 µg) than in the placebo group. The findings showed low and moderate variability and were consistent across trials. The random effects model’s wide confidence intervals showed that the estimates varied. The results’ robustness was reinforced by the effect size and consistency of the findings (**Figure 8**). Also, headaches are more common in the treatment group (ROSE-010 at 300 µg) than in the placebo group. The treatment group has an increased probability of experiencing headaches, according to the odds ratios (OR) from the random effects model (2.75) and the common effect model (3.07). However, the estimates’ variability and degree of uncertainty are indicated by the broad confidence intervals, especially in the random effects model (**Figure 9**).

## Discussion

It is challenging to formulate effective treatment options for IBS since the symptom likely represents a variety of medical conditions (36). One of the main symptoms of IBS and the primary explanation for why patients seek medical attention is abdominal pain related to bowel movements (37, 38). Although there are a number of well-established management solutions, their availability varies, and their use is not always ideal (39). For vulnerable individuals experiencing extreme pain, it is necessary to avoid iatrogenesis and uncontrolled, inadequately supported methods. Although there is a promising pipeline of new preclinical and early clinical targets and management strategies, there are still a lot of unanswered questions; thus, pain in IBS continues to be a focus for quality improvement and research (40).

Our systematic analysis of GLP-1 agonist’s impact on IBS-related pain and symptoms suggests a promising landscape for its use beyond standard metabolic applications. GLP-1 receptor agonists’ multifaceted therapeutic properties extend their benefits to a wide range of pain disorders, including inflammatory, visceral, and neuropathic pain (41, 42). This meta-analysis demonstrated that ROSE-010 significantly lowers pain intensity in patients with IBS compared to a placebo. The odds ratios for the 100 µg and 300 µg doses show a significant positive effect. The low variability across trials increases the reliability of these findings. The meta-analysis supports ROSE-010’s effectiveness in treating IBS symptoms.

Previous studies provided comparative results and evidence that ROSE-010 is effective in controlling pain and improving gastrointestinal motility in IBS patients. Hellström et al. (2009) conducted a placebo-controlled experiment to assess the effectiveness of ROSE-010 in relieving pain in all IBS subtypes. The trial included two doses. The results showed that at one hour post-treatment, the ROSE-010 groups had twice as many pain alleviation responders as the placebo group. The study additionally showed that ROSE-010 was most helpful at relieving pain in IBS-C and IBS-M (24). Touny et al. (2022) conducted a cross-analysis to assess the pain alleviation and intensity responses to ROSE-010 in IBS patients. The trial involved 166 subjects and concluded that ROSE-010 provided dosage- and time-dependent pain alleviation, with the highest relief obtained at the 300 µg dose. Females reported more pain reduction than males, and the drug was more beneficial for IBS-C and IBS-M (12). Halloum et al. (2024) conducted a systematic review to assess the efficacy of GLP-1 receptor agonists, such as ROSE-010, for headache and pain concerns. The review stated that GLP-1 receptor agonists have analgesic properties by modifying pain hypersensitivity in animal models of inflammation and neuropathic pain. The review also found that GLP-1 receptor agonists diminish visceral hypersensitivity and alleviate symptoms in IBS patients (43).

GLP-1 agonists, such as ROSE-010, relieve pain in IBS patients by altering gut motility (43), lowering inflammation (44), modulating neurotransmitters (45), reducing visceral hypersensitivity (46), and interacting with the gut microbiota (47). These pathways help to explain GLP-1 agonists’ pain-relieving benefits in IBS patients. They can help reduce visceral hypersensitivity, a frequent symptom in IBS (48, 49). Following meal consumption, the metabolic endocrine cascade begins with gastric emptying. Insulin secretion is stimulated, glucagon and gastric acid secretion are inhibited, and GI transit and motility are decreased by incretin hormones, especially GLP-1, which is released postprandially from L-cells lining the gut in response to food ingestion (50–52). Furthermore, both *in vitro* and *in vivo*, GLP-1 has been frequently demonstrated to reduce GI muscle activity via nerve-mediated mechanisms reliant on nitric oxide (13, 53). In addition to being expressed in peripheral tissues, the GLP-1 receptor is also expressed in the central nervous system (CNS) and is limited to neurons in the caudal nucleus of the solitary tract (NTS) and the ventrolateral medulla in the brainstem and hypothalamus (54, 55). Biologically active GLP-1 has a high affinity for this receptor (56, 57). Additionally, the GLP-1 receptors has been identified in the GI tract’s myenteric and submucosal neural plexuses (55). GLP-1 inhibited the migrating motor complex (MMC) and decreased motility in the antro-duodeno-jejunal region in both healthy subjects and IBS patients (22).

Constipation-predominant IBS was linked to lower mucosal expression of GLP-1R and blood GLP-1 concentrations (28). It is believed that reduced concentrations of GLP-1 would result in loss of the pro-kinetic actions of GLP-1 in the colon [Citation26], causing constipation and abdominal discomfort, was further supported by the correlation between this and the intensity of abdominal pain (12). A rat model of visceral pain sensitivity also showed a reduction in circulating amounts of bioactive GLP-1 (58). GLP-1 and ROSE-010 suppress postprandial GI motility, most likely via GLP-1R at myenteric neurons, necessitating functioning cAMP and nitrergic signaling (13).

According to the current study’s findings, ROSE-010 is linked to a greater incidence of headaches, nausea, and vomiting than placebo. These adverse effects are probably caused by the drugs themselves rather than by extraneous factors, as indicated by the results’ consistency across several studies and low degree of heterogeneity. It seems that ROSE-010 has demonstrated encouraging outcomes in treating acute pain in people with IBS, as compared to earlier research. But of particular concern is the increase in headaches, nausea, and vomiting. Adverse effects have been documented in prior studies (12, 21, 24), suggesting that although ROSE-010 is useful in treating pain, its tolerance profile should be carefully evaluated.

Several medications have been approved for the management of IBS, each targeting a particular mechanism and use for a specific disease subtype. In contrast to pure μ-opioid agonists, eluxadoline, a little-absorbed combined μ- and κ-opioid receptor agonist and δ-opioid receptor antagonist, was developed to decrease constipation and boost analgesic effectiveness (59, 60). The FDA has authorized 100 mg of eluxadoline twice daily for the treatment of IBS-D. Patients with mild to severe hepatic impairment, those using concurrent OATP1B1 inhibitors, or those who can’t tolerate the 100 mg dosage are advised to use eluxadoline 75 mg twice daily (61). Constipation (8%), nausea (7%), and stomach pain (7%), among individuals receiving eluxadoline, were the most frequent side effects (61). Gram-positive and gram-negative anaerobic and aerobic bacteria are both susceptible to the wide-ranging effects of the nonabsorbable oral antibiotic rifaximin (62). The FDA has approved a dose of 550 mg three times a day for 14 days to treat IBS-D. Using the same dosing schedule, patients can receive treatment up to twice if their symptoms return (63). Nasopharyngitis, urinary tract infections, upper respiratory infections, and nausea were the most frequent side effects (64).

The mechanism of action of the selective 5-HT3 antagonist alosetron is thought to be both centrally and peripherally mediated (65). The FDA first authorized Alosetron in 2000 to treat IBS-D in women. However, it was voluntarily discontinued because of severe side effects, including ischemic colitis and severe constipation problems (66). Alosetron’s reintroduction was authorized by the FDA in 2002, but it was limited to treating women with severe IBS-D as part of a risk-management program (67). Half mg twice daily is the first suggested starting dose. Patients who have constipation should stop taking the drug until their symptoms relieve. They can resume taking 0.5 mg once daily, but alosetron should be stopped if constipation returns at a lower dosage. After four weeks, the dose can be raised to 1 mg twice a day if symptoms are still uncontrollable. Alosetron should be stopped after 4 weeks if symptoms still exist, even if the dosage is increased to 1 mg twice a day (61).

In clinical practice, antispasmodics are frequently used for alleviating IBS-related abdominal pain. Despite being a pharmacologically varied class, antispasmodics are believed to alleviate the symptoms of IBS by lowering visceral hypersensitivity and smooth muscle contraction (68). The only antispasmodics that are accessible in the US are peppermint oil, hyoscine, and dicyclomine.

Compared to the previously discussed side effects of approved IBS medications, ROSE-010 is typically well tolerated; however, adverse events (AEs) can occur. The expected medication effects, which include nausea, vomiting, and headache, are rarely severe (21, 24).

The current study has certain limitations. The meta-analysis, based on a limited sample size of five studies, may impact the statistical power and generalizability of the findings. The restricted number of studies also raises issues about the representativeness of the findings. Future research should prioritize larger, well-designed studies to increase statistical power, reliability, and generalizability. Subgroup analysis according to IBS subtypes (e.g., IBS-C, IBS-D, IBS-M, and IBS-U) would have offered significant insight on how the intervention differed in its effects on these various patient groups. However, such subgroup analyses were not possible because of the small number of studies. Given that there are several subtypes of IBS and that the condition’s etiology and symptom profiles vary, this is another limitation of the study. Furthermore, the study’s absence of comparisons to other medications may make it difficult to implement ROSE-010 in the current therapy landscape. As a result, more clinical trials are needed to better understand ROSE-010’s potential for IBS management. The suggested clinical trial pipeline involves longitudinal investigations, randomized controlled trials, post-marketing surveillance, as well as health economic analysis. It attempts to address the knowledge gaps and give a comprehensive understanding of the treatment’s long-term effects, resulting in better clinical decisions and patient outcomes.

## Conclusion

The glucagon-like peptide-1 receptor agonist ROSE-010 has been shown in studies to be beneficial in lowering the severity of pain in individuals with IBS. The drug’s reliability is demonstrated by the findings, which are consistent throughout several trials. However, the higher frequency of headaches, nausea, and vomiting at the 100 µg dosage emphasizes the necessity of close monitoring. The study also highlights the value of individualized treatment plans that modify therapy according to the unique needs of each patient. The impact on various IBS subtypes, methods of action, and long-term effects require more investigation. Clinical recommendations and guidelines for the use of ROSE-010 in the treatment of IBS may be influenced by the findings of this study. To maximize outcomes, healthcare professionals must balance the advantages against the possibility of side effects and take individualized treatment modalities into account.

## Data Availability

The original contributions presented in the study are included in the article/supplementary material. Further inquiries can be directed to the corresponding author.
